# Levodopa inhibits the development of lens-induced myopia in chicks

**DOI:** 10.1038/s41598-020-70271-z

**Published:** 2020-08-06

**Authors:** Kate Thomson, Ian Morgan, Cindy Karouta, Regan Ashby

**Affiliations:** 1 Centre for Research into Therapeutic Solutions, Faculty of Science and Technology, University of Canberra, Canberra, Australia; 2 Research School of Biology, Australian National University, Canberra, Australia

**Keywords:** Retina, Neurochemistry, Receptor pharmacology, Drug delivery

## Abstract

Animal models have demonstrated a link between dysregulation of the retinal dopamine system and the development of myopia (short-sightedness). We have previously demonstrated that topical application of levodopa in chicks can inhibit the development of form-deprivation myopia (FDM) in a dose-dependent manner. Here, we examine whether this same protection is observed in lens-induced myopia (LIM), and whether levodopa’s protection against FDM and LIM occurs through a dopamine D1- or D2-like receptor mechanism. To do this, levodopa was first administered daily as an intravitreal injection or topical eye drop, at one of four ascending doses, to chicks developing LIM. Levodopa’s mechanism of action was then examined by co-administration of levodopa injections with D1-like (SCH-23390) or D2-like (spiperone) dopamine antagonists in chicks developing FDM or LIM. For both experiments, levodopa’s effectiveness was examined by measuring axial length and refraction after 4 days of treatment. Levodopa inhibited the development of LIM in a dose-dependent manner similar to its inhibition of FDM when administered via intravitreal injections or topical eye drops. In both FDM and LIM, levodopa injections remained protective against myopia when co-administered with SCH-23390, but not spiperone, indicating that levodopa elicits its protection through a dopamine D2-like receptor mechanism in both paradigms.

## Introduction

Myopia, commonly known as short-sightedness, is a refractive disorder arising from a mismatch between the axial length and optical power of the eye. This is generally due to excessive elongation of the eye during development and into early adulthood. In urban East and Southeast Asia the prevalence of myopia in young adults has risen from 20–30% to 80–85% in the last five decades (For review see^1^). The prevalence of high myopia (≤ − 6 diopters (D)) has increased disproportionately to that of myopia in the last five decades, rising from 1–5% to 10–20% (For review see^1^). Although the refractive error associated with this condition can easily be corrected, such corrections do not address the sight-threatening pathologies associated with myopia, and especially high myopia, which include retinal detachment, myopic macular degeneration, staphyloma, glaucoma, and cataracts^2–6^. Furthermore, the odds of such pathologies significantly increase with the severity of myopia^7^.

Through work in animal models, changes in retinal dopamine release have been heavily implicated in the development of myopia (for review see^8–10^). Specifically, in chicks, rhesus monkeys, guinea pigs, tree shrews and in some cases mice, retinal levels of dopamine, and its primary metabolite 3,4-dihydroxyphenylacetic acid (DOPAC), have been shown to be significantly down-regulated during the development of form-deprivation myopia (FDM)^11–16^. Consistent with a role for dopamine in the regulation of ocular growth, administration of dopaminergic agonists^11,17–23^, synthetic dopamine^24^, and its metabolic precursor, levodopa^25–28^, inhibit FDM. Conversely, retina-specific tyrosine hydroxylase knockout mice and mice treated with 6-hydroxydopamine, which depletes the retina of dopaminergic neurons, show a myopic shift in refraction^15,29^. Furthermore, the ability of bright light to inhibit FDM through increased dopamine release^30–33^ is inhibited by the administration of dopaminergic antagonists in chicks and mice^34,35^. Similarly, the protective effects of brief periods of normal vision against the development of FDM in chicks is lost by modulating dopaminergic function by either keeping the animals in the dark during diffuser removal, thus inhibiting dopamine release, or by injecting a dopaminergic D2-like receptor antagonist^19^.

Together, these findings suggest that a depletion in retinal dopamine release is associated with myopic eye growth. In accordance with this hypothesis, we have previously shown in chicks that topical or intravitreal application of the dopamine precursor, levodopa, can increase retinal dopamine release and inhibit the development of FDM in a dose-dependent manner^27^. To expand on our previous work in FDM, we investigate here whether this same dose-dependent protection can be generated in chicks undergoing lens-induced myopia (LIM), as the role of dopamine in this experimental model is less clear^36–38^. Specifically, although the physiological changes seen in response to FDM and LIM are similar, due to distinctions in the way in which myopia is induced between these two paradigms, there are potential differences in the underlying retinal mechanism driving growth. FDM is induced by placing a translucent diffuser in front of the eye to deprive it of form-vision and thereby cause myopic growth^39,40^. As the eye never compensates for this form of defocus, ocular growth will continue for as long as the diffuser remains attached and developmental plasticity remains. This is therefore referred to as an open-loop system, as there is no specific end-point for growth. In contrast, LIM is induced by placing a negative (concave) lens in front of the eye, which pushes the focal plane behind the retina and encourages the eye to elongate to compensate for this imposed defocus^41,42^. This is referred to as a closed-loop system as there is a defined end-point, with eye growth returning to normal growth rates once compensation to the imposed defocus is achieved.

To pharmacologically test whether levodopa does indeed inhibit experimental myopia by increasing retinal dopamine synthesis and release^27^, here we also examine whether administration of dopaminergic antagonists can prevent levodopa from inhibiting experimental myopia. Dopaminergic activation is mediated through 5 major subtypes of G-protein coupled receptors which are divided into two families, D1-like (D_1_ and D_5_ receptors) and D2-like (D_2_, D_3_ and D_4_ receptors) (for review see^43^), both of which are expressed in the chicken retina^44,45^. Work undertaken in chicks^11,19–21,34,46^ and tree shrews^22^ has demonstrated a D2-dependent mechanism for the dopaminergic inhibition of experimental myopia in these models. However, this does not appear to be consistent across all species, with members of the Rodentia family (guinea pigs^47^ and mice^48^) demonstrating protection through a D1-like receptor mechanism. Therefore, we examine whether levodopa’s protective effects are blocked by co-administration with either the D1-like receptor antagonist SCH-23390 or the D2-like receptor antagonist spiperone.

## Results

### Levodopa inhibits the development of LIM in a dose-dependent manner

To establish whether the dose-dependent protective effects of levodopa against the development of FDM are preserved in LIM, chicks were treated with one of four ascending doses of levodopa (Table 1), administered as either a once-daily intravitreal injection (to directly target the retina) or twice-daily topical eye drops (to represent a more clinically-relevant avenue for treatment), for a period of 4 days.

**Table 1:** Dosages and conditions for drug solutions used.

Drug	Application avenue	Ocular treatment	Concentration (mM)	Concentration (% w/v)	Treatments per day	Volume given daily (µL)	Amount given (mg/day)	Amount given (mg/kg/day)	Number of animals
Levodopa	Drops	Lens	0.15	0.003	2	160	0.005	0.038	8
Levodopa	Drops	Lens	1.50	0.030	2	160	0.047	0.379	8
Levodopa	Drops	Lens	15.00	0.296	2	160	0.473	3.786	7
Levodopa	Drops	Lens	45.00	0.887	2	160	1.420	11.358	8
Vehicle Solution	Drops	Lens	n/a	n/a	2	160	n/a	n/a	8
Levodopa	Injection	Lens	0.15	0.003	1	10	0.0003	0.002	6
Levodopa	Injection	Lens	1.50	0.030	1	10	0.003	0.024	6
Levodopa	Injection	Lens	15.00	0.296	1	10	0.030	0.237	6
Levodopa	Injection	Lens	45.00	0.887	1	10	0.090	0.711	6
Vehicle Solution	Injection	Lens	n/a	n/a	1	10	n/a	n/a	8
Levodopa	Injection	Lens	15.00	0.296	1	10	0.030	0.237	6
Levodopa/SCH-23390	Co-injection	Lens	15.00/0.50	0.296/0.014	1	10	0.030/0.0014	0.240/0.0112	6
Levodopa/Spiperone	Co-injection	Lens	15.00/0.50	0.296/0.002	1	10	0.030/0.0002	0.240/0.0016	6
Levodopa	Injection	Diffuser	15.00	0.296	1	10	0.030	0.237	6
Levodopa/SCH-23390	Co-injection	Diffuser	15.00/0.50	0.296/0.014	1	10	0.030/0.0014	0.240/0.0112	6
Levodopa/Spiperone	Co-injection	Diffuser	15.00/0.50	0.596/0.002	1	10	0.030/0.0002	0.240/0.0016	6

For all treatments, there was no significant difference in axial length (p = 0.607) or refraction (p = 0.545) between contralateral control eyes and age-matched untreated control eyes at the end of the treatment period. LIM (-10D) induced a significantly greater rate of axial growth and a significant myopic shift in refraction in treated eyes relative to contralateral control (axial p < 0.001, refraction p < 0.001) and age-matched untreated control animals (Table 2). Treatment with the vehicle solution (0.1% ascorbic acid in 1×PBS) did not alter the development of LIM when administered as either an intravitreal injection or topical eye drops (Table 2).

**Table 2:** Axial length and refractive measurements for levodopa LIM dose–response curves.

Condition	Axial length				Refraction			
	Left eye	Right eye	Compared to LIM	Compared to untreated	Left eye	Right eye	Compared to LIM	Compared to untreated
Untreated	8.69 ± 0.04	8.71 ± 0.03	**p < 0.001**	–	2.17 ± 0.20	2.30 ± 0.16	**p < 0.001**	–
LIM Only	9.11 ± 0.06	8.68 ± 0.03	–	**p < 0.001**	− 1.80 ± 0.15	2.14 ± 0.21	–	**p < 0.001**
LIM Vehicle Injections	8.97 ± 0.04	8.68 ± 0.02	p = 0.503	**p < 0.001**	− 1.23 ± 0.28	2.33 ± 0.16	p = 0.674	**p < 0.001**
LIM Vehicle Drops	9.08 ± 0.05	8.68 ± 0.04	p = 0.834	**p < 0.001**	− 1.35 ± 0.22	2.68 ± 0.11	p = 0.823	**p < 0.001**
**LIM + levodopa intravitreal injections**								
0.15 mM	8.85 ± 0.04	8.66 ± 0.03	p = 0.076	p = 0.189	− 0.62 ± 0.40	2.12 ± 0.17	**p = 0.039**	**p < 0.001**
1.5 mM	8.81 ± 0.06	8.67 ± 0.06	**p = 0.026**	p = 0.665	0.22 ± 0.17	2.30 ± 0.19	**p < 0.001**	**p = 0.001**
15 mM	8.71 ± 0.05	8.62 ± 0.04	**p = 0.001**	p = 1.000	0.80 ± 0.5	2.30 ± 0.40	**p < 0.001**	p = 0.081
45 mM	8.75 ± 0.07	8.64 ± 0.05	**p = 0.005**	p = 1.000	0.50 ± 0.12	2.10 ± 0.18	**p < 0.001**	p = 0.056
**LIM + Levodopa Drops**								
0.15 mM	8.91 ± 0.04	8.66 ± 0.04	p = 0.198	**p = 0.017**	− 0.80 ± 0.21	2.45 ± 0.19	**p = 0.013**	**p < 0.001**
1.5 mM	8.87 ± 0.05	8.68 ± 0.04	**p = 0.048**	p = 0.094	− 0.62 ± 0.19	2.20 ± 0.15	**p = 0.002**	**p < 0.001**
15 mM	8.86 ± 0.06	8.67 ± 0.03	**p = 0.048**	p = 0.157	− 0.30 ± 0.25	2.04 ± 0.12	**p < 0.001**	**p < 0.001**
45 mM	8.83 ± 0.06	8.63 ± 0.04	**p = 0.015**	p = 0.303	− 0.31 ± 0.18	2.30 ± 0.22	**p < 0.001**	**p < 0.001**

Data are presented as the mean ± standard error of the mean, statistics are presented as pairwise comparisons with Bonferroni correction, with significant comparisons (p < 0.05) presented in bold.

*LIM* Lens induced myopia, *Untreated* age-matched untreated controls.

**Figure 1:**
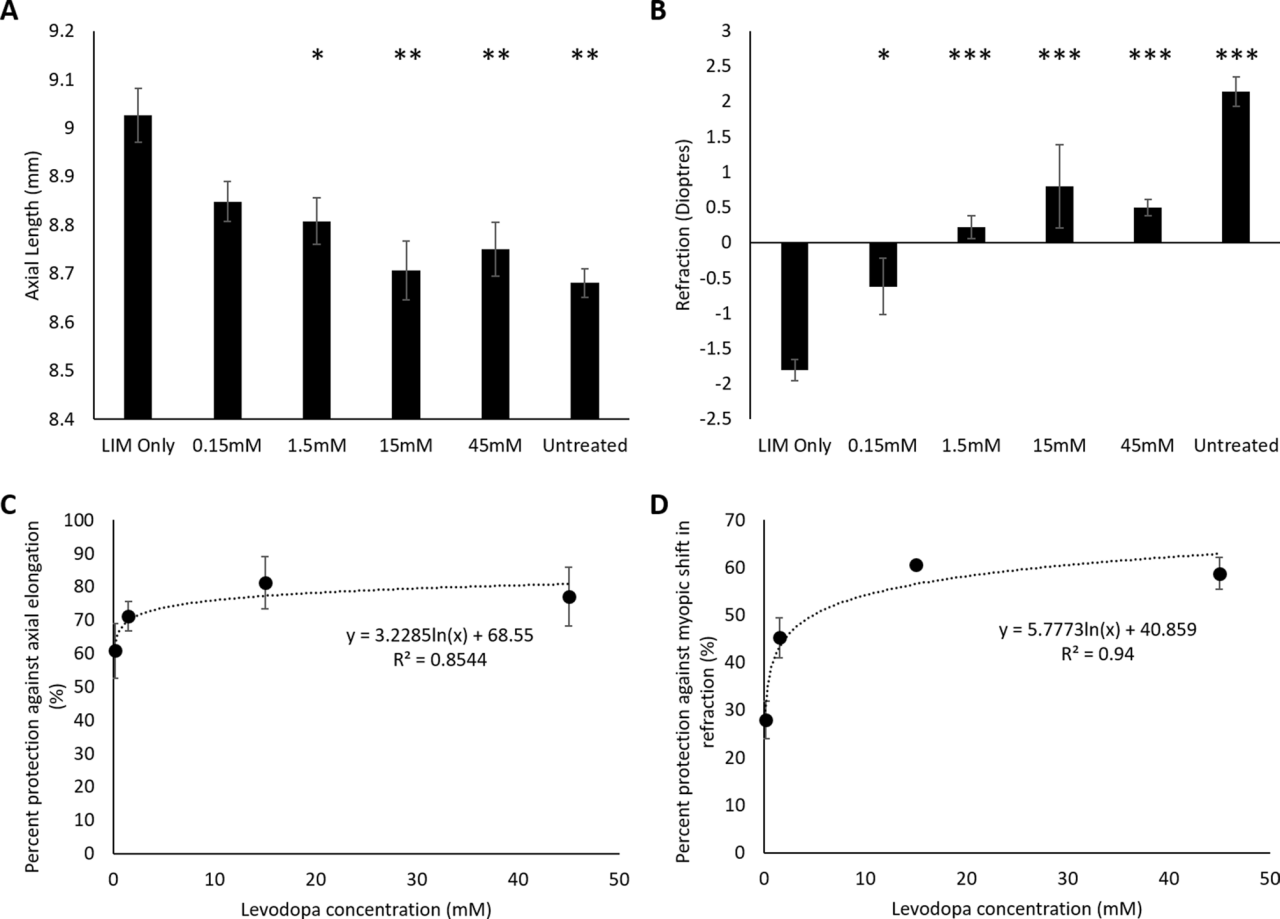
Dose-response curve: levodopa intravitreal injections into negative lens-treated eyes. (**A**) Axial length measurements; (**B**) refraction measurements; (**C**) percent protection against the axial elongation associated with LIM; (**D**) percent protection against the myopic shift in refraction associated with LIM. Data represents the mean ± standard error of the mean. *LIM* Lens-induced myopia, *Untreated* Age-matched untreated controls. Concentrations stated represent the concentration of levodopa administered. Statistics denote differences between levodopa treated eyes and LIM only; *p < 0.05, **p < 0.01, ***p < 0.001.

Daily intravitreal injections of levodopa significantly inhibited both the excessive axial elongation (Fig. 1A, Tables 2 and 3) and myopic refractive shift (Fig. 1B, Tables 2 and 3) associated with LIM. This dose-dependent protection was best described by a logarithmic relationship for both axial length (y = 3.2285In(x) + 68.55, r^2^ = 0.8544; EC_50_ = 0.003 mM (0.00006% w/v, 0.000006 mg/day); Fig. 1C) and refraction (y = 5.7773ln(x) + 40.859, r^2^ = 0.94; Fig. 1D). Although a significant difference in axial length and refraction remained between levodopa treated chicks and age-matched untreated controls (Table 3), at doses 15 mM and above there was no statistically significant difference in axial length or refraction between treated and age-matched untreated control eyes (Table 2). Levodopa treatment did not induce changes in anterior chamber depth or lens thickness, but rather levodopa’s protection was elicited by inhibiting vitreal chamber elongation (Table 3).

**Table 3:** ANOVA comparisons for ocular biometry and refractive measurements of levodopa LIM dose–response curves.

Condition	Ocular biometry					Refraction	
	Axial length compared to LIM only	Axial length compared to untreated	Anterior chamber depth compared to LIM only	Lens thickness compared to LIM only	Vitreal chamber depth compared to LIM only	Compared to LIM only	Compared to untreated
LIM + Levodopa Intravitreal Injections	**F(4, 28) = 7.810, p = 0.001**	**F(4, 28) = 2.918, p = 0.046**	F(4, 28) = 2.322, p = 0.090	F(4, 28) = 1.904, p = 0.147	**F(4, 28) = 5.693, p = 0.003**	**F(4, 28) = 15.464, p < 0.001**	**F(4,28) = 14.408, p < 0.001**
LIM + Levodopa Drops	**F(4, 35) = 3.841, p = 0.011**	**F(4, 35) = 3.394, p = 0.019**	F(4, 35) = 0.737, p = 0.539	F(4, 35) = 1.496, p = 0.238	**F(4, 35) = 4.512, p = 0.006**	**F(4, 35) = 11.402, p < 0.001**	**F(4, 35) = 36.563, p < 0.001**

Significant comparisons (p < 0.05) are presented in bold.

*LIM* lens induced myopia.

**Figure 2:**
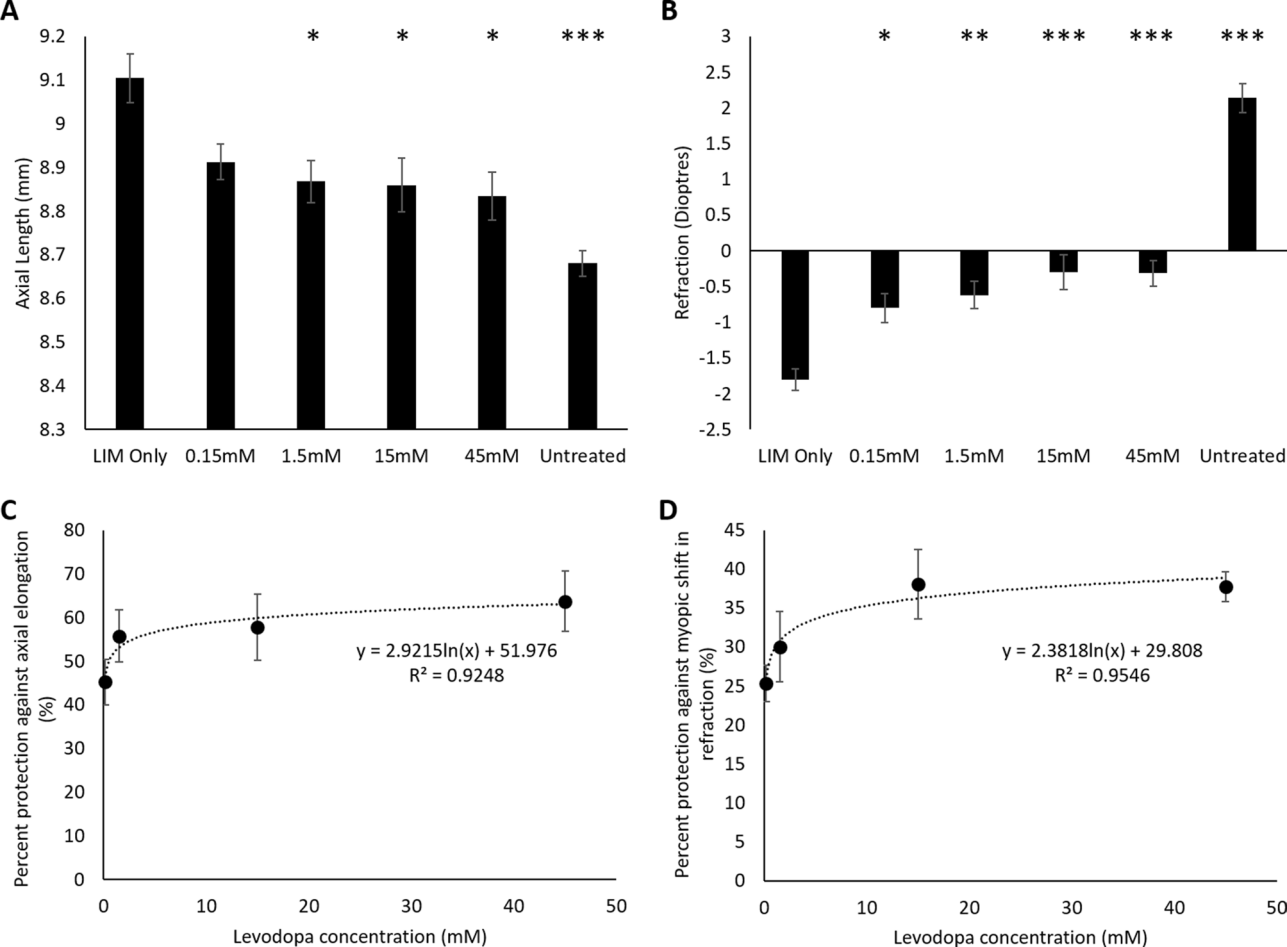
Dose-response curve: levodopa topical eye drops into negative lens-treated eyes. (**A**) Axial length measurements; (**B**) refraction measurements; (**C**) percent protection against the axial elongation associated with LIM; (**D**) percent protection against the myopic shift in refraction associated with LIM. Data represents the mean ± standard error of the mean. *LIM* Lens-induced myopia, *Untreated* Age-matched untreated controls. Concentrations stated represent the concentration of levodopa administered. Statistics denote differences between levodopa treated eyes and LIM only; *p < 0.05, **p < 0.01, ***p < 0.001.

Similarly, daily treatment with topical levodopa eye drops also inhibited both the excessive ocular growth (Fig. 2A, Tables 2 and 3) and negative shift in refraction (Fig. 2B, Tables 2 and 3) associated with LIM. Once again this dose-dependent protection afforded by levodopa was best described by a logarithmic relationship for both axial length (y = 2.9215In(x) + 51.98, r^2^ = 0.9248; EC_50_ = 0.51 mM (0.01% w/v, 0.02 mg/day); Fig. 2C) and refraction (y = 2.3818ln(x) + 29.808, r^2^ = 0.9546; Fig. 2D). However, even at the highest dose, full protection against LIM was not observed, with a significant difference in both axial length and refraction remaining between LIM/levodopa treated eyes and age-matched untreated control eyes (Table 3), with this difference also observed between levodopa treated and contralateral control eyes (axial: p = 0.230, refraction: p = 0.421). Levodopa treatment did not induce changes in anterior chamber depth or lens thickness, its protection was again elicited by slowing vitreal chamber elongation (Table 3).

Although no change in lens thickness or anterior chamber depth was observed at any concentration of levodopa, to confirm that topical levodopa did not affect the optical power of the eye, corneal curvature was measured in the 15 mM topical levodopa group. Levodopa treatment demonstrated no effects on corneal curvature (levodopa treated eyes 3.19 ± 0.04 mm radius of curvature vs contralateral control eyes 3.25 ± 0.06 mm radius of curvature; p = 0.512), or lens power when calculated using Bennet’s equation, adjusted for chicks^49^ (levodopa treated eyes 75.72 ± 2.17 dioptres vs contralateral control eyes 71.45 ± 3.66 dioptres; p = 0.091).

### Levodopa treatment inhibits the development of LIM in a similar dose-dependent manner to that of FDM

**Figure 3:**
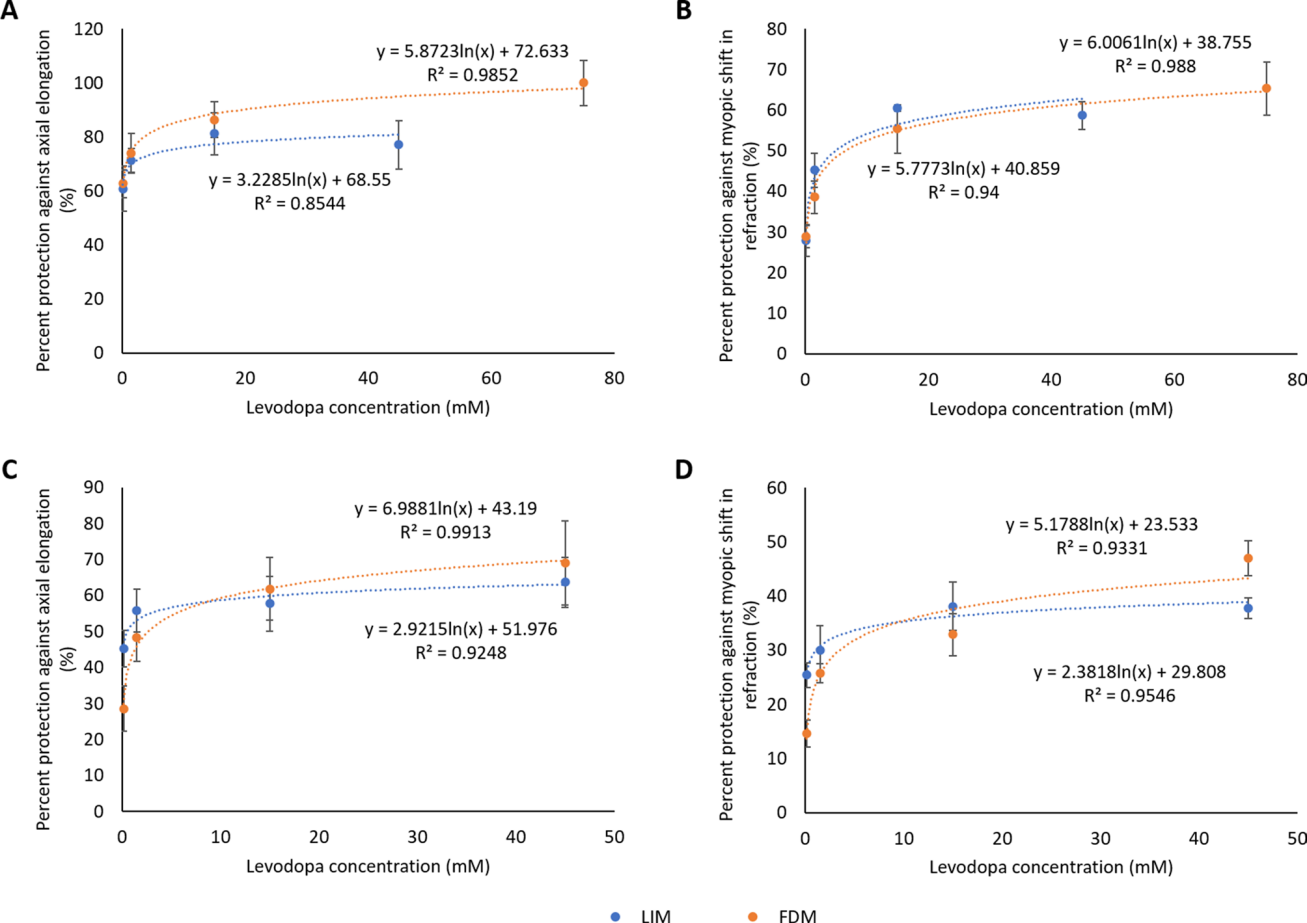
Comparison of dose–response curves between FDM and LIM. LIM (blue) levodopa dose–response curves were compared to FDM (orange) levodopa dose–response curves from previous data. (**A**) Percent protection of intravitreal levodopa against axial elongation; (**B**) percent protection of intravitreal levodopa against myopic refractive shifts; (**C**) percent protection of topical levodopa against axial elongation; (**D**) percent protection of topical levodopa against myopic refractive shifts. *LIM* lens-induced myopia, *FDM* form-deprivation myopia. Data represents the mean ± standard error of the mean. Data plotted for FDM are drawn from a previous publication for comparison^27^.

To compare the effectiveness of levodopa treatment between FDM and LIM, the dose-dependent effects of levodopa in negative-lens treated eyes were retrospectively compared to previous data on the dose-dependent effects of levodopa in FDM eyes^27^ treated following the same methodology and within the same developmental timeframe. There was no significant difference in axial length (p = 0.151) or refraction (p = 0.572) between FDM only and LIM (-10D) only chicks after 4 days of treatment, thus both paradigms showed a similar degree of myopia development over this brief timeframe. Intravitreal injection of levodopa inhibited the development of LIM in a dose-dependent manner similar to that previously seen for FDM^27^. Specifically, neither axial length (Wilk’s Lambda = 0.759, F(1,55) = 0.955, p = 0.455; Fig. 3A), nor refraction (Wilk’s Lambda = 0.557, F(1,55) = 2.386, p = 0.137; Fig. 3B) measurements were significantly different between the two forms of experimental myopia over the dose range tested. Similarly, topical application of levodopa showed a dose-dependent inhibition of LIM that was indistinguishable from that seen for FDM^27^ (axial length (Wilk’s Lambda = 0.787, F(1,89) = 0.814, p = 0.540; Fig. 3C); refraction (Wilk’s Lambda = 0.673, F(1,89) = 4.000, p = 0.059; Fig. 3D)).

### Levodopa elicits its protective effects through a D2-like dopamine receptor dependent mechanism

**Figure 4:**
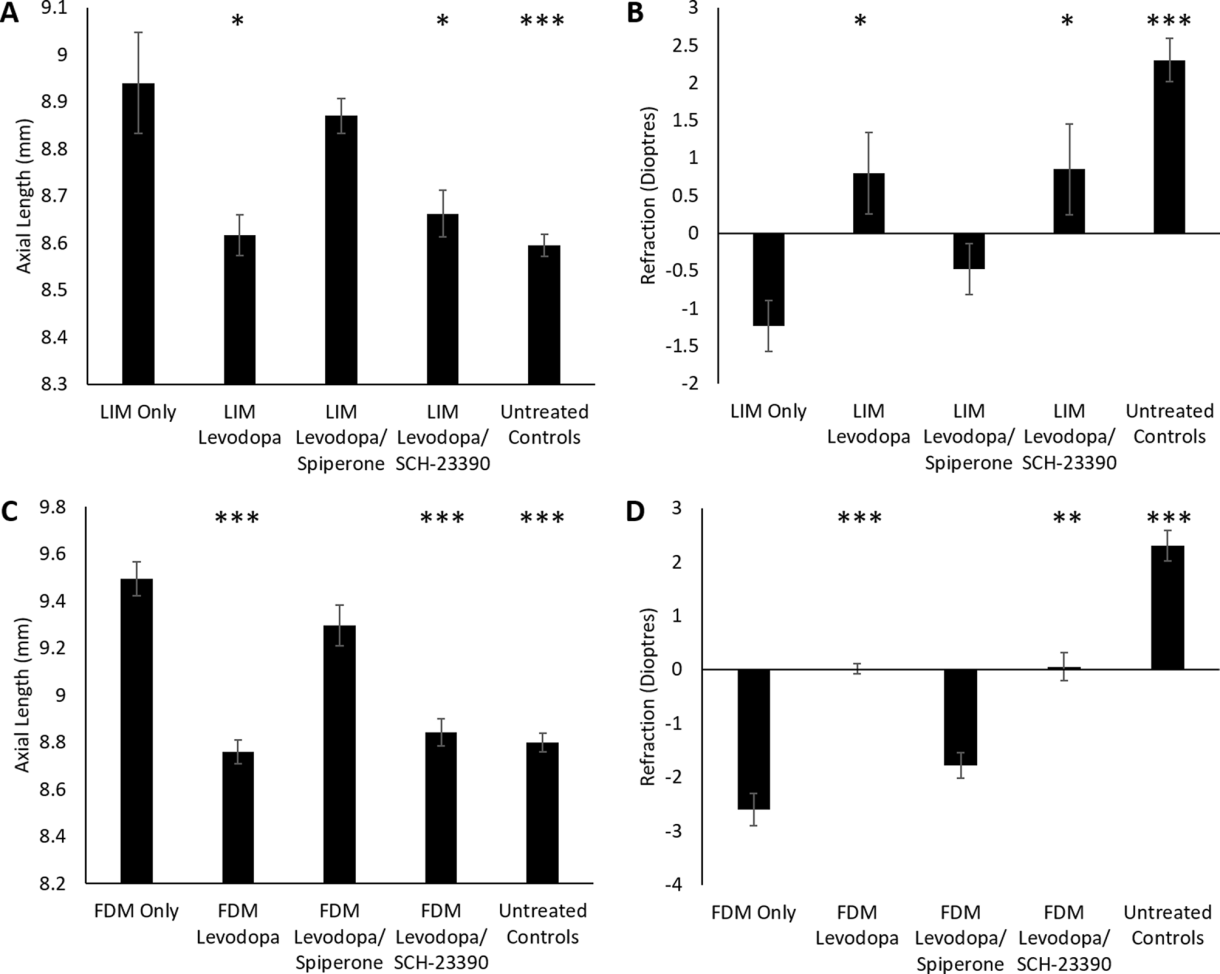
Co-administration of levodopa with dopaminergic antagonists. (**A**) Axial length of LIM chicks; (**B**) refraction of LIM chicks; (**C**) axial length of FDM chicks; (**D**) refraction of FDM chicks. Data represents the mean ± standard error of the mean. Concentration of drug administered: levodopa (15 mM), spiperone (0.5 mM) and SCH-23390 (0.5 mM). Statistics denote difference to myopia; *p < 0.05, **p < 0.01, ***p < 0.001. *FDM* Form-deprivation myopia, *LIM* Lens-induced myopia, *Untreated* Age-matched untreated controls.

To establish the receptor subtype by which levodopa induced dopamine release inhibits experimental myopia, intravitreal injections of 15 mM levodopa were co-administered with either an antagonist of the D1-like dopamine receptor family (SCH-23390) or an antagonist of the D2-like dopamine receptor family (spiperone) to chicks undergoing FDM or LIM for a period of 4 days. As seen above, 15 mM levodopa injections significantly inhibited the excessive axial elongation associated with LIM (Fig. 4A, Table 4). This protective effect against the axial elongation associated with LIM persisted when levodopa was co-injected daily with the D1-like dopamine receptor antagonist SCH-23390 over the four-day treatment period, however, was lost when levodopa was co-injected with the D2-like dopamine receptor antagonist spiperone, leaving chicks no different to LIM only animals. A similar trend was seen in refraction (Fig. 4B, Table 4), with levodopa only and levodopa/SCH-23390 injections inhibiting the myopic refractive shift associated with LIM, whilst levodopa/spiperone treated eyes were not statistically different to LIM only eyes.

**Table 4:** Axial length and refraction measurements for co-administration of levodopa with dopaminergic antagonists**.**

Condition	Axial length				Refraction			
	Left eye	Right eye	Compared to myopia	Compared to untreated	Left eye	Right eye	Compared to myopia	Compared to untreated
Untreated	8.75 ± 0.04	8.72 ± 0.04	**p < 0.001**	**–**	2.10 ± 0.30	2.34 ± 0.40	**p < 0.001**	**–**
FDM only	9.49 ± 0.07	8.79 ± 0.03	**–**	**p < 0.001**	− 2.60 ± 0.20	2.20 ± 0.20	**–**	**p < 0.001**
LIM only	8.94 ± 0.10	8.71 ± 0.05	**–**	**p < 0.001**	− 1.23 ± 0.30	1.80 ± 0.30	**–**	**p < 0.001**
**FDM + levodopa**								
Levodopa	8.76 ± 0.05	8.70 ± 0.05	**p < 0.001**	p = 0.793	1.04 ± 0.23	2.71 ± 0.11	**p < 0.001**	**p = 0.023**
Levodopa/SCH-23390	8.84 ± 0.05	8.76 ± 0.03	**p = 0.009**	p = 0.322	0.05 ± 0.26	2.62 ± 0.05	**p = 0.002**	**p = 0.009**
Levodopa/Spiperone	9.30 ± 0.09	8.75 ± 0.05	p = 0.106	**p < 0.001**	− 1.78 ± 0.23	2.66 ± 0.03	p = 0.129	**p < 0.001**
**LIM + levodopa**								
Levodopa	8.71 ± 0.05	8.62 ± 0.04	**p = 0.001**	p = 1.000	0.80 ± 0.50	2.30 ± 0.40	**p < 0.001**	p = 0.081
Levodopa/SCH-23390	8.66 ± 0.05	8.73 ± 0.07	**p = 0.048**	p = 0.568	0.85 ± 0.60	2.53 ± 0.20	**p = 0.021**	p = 0.345
Levodopa/Spiperone	8.85 ± 0.05	8.60 ± 0.02	p = 0.521	**p = 0.045**	− 0.48 ± 0.30	2.54 ± 0.02	p = 0.409	**p < 0.001**

Data are presented as the mean ± standard error, statistics are presented as pairwise comparisons with Bonferroni correction, with significant comparisons (p < 0.05) presented in bold. Concentration of drug administered: levodopa (15 mM), spiperone (0.5 mM) and SCH-23390 (0.5 mM).

*FDM* Form-deprivation myopia, *LIM* Lens-induced myopia, *Untreated* age-matched untreated controls.

The same effect was observed for FDM, with the axial elongation associated with diffuser-wear inhibited by 15 mM levodopa injections (Fig. 4C, Table 4) and levodopa/SCH-23390 injections, but not levodopa/spiperone injections, and the myopic shift observed with FDM inhibited by levodopa (Fig. 4D, Table 4) and levodopa/SCH-23390, but not levodopa/spiperone.

## Discussion

Intravitreal and topical application of levodopa slowed ocular growth and significantly inhibited the development of lens-induced myopia (LIM) in a dose-dependent manner. Levodopa retarded the development of LIM by inhibiting the rate of vitreal chamber elongation without affecting the optical power of the eye as no change was observed in corneal radius of curvature, anterior chamber depth, lens thickness or lens power following four-days of treatment.

Intravitreal injection inhibited LIM to a greater extent than that of topical application. This is not unexpected as typically less than 3% of a topically applied compound reaches the posterior portion of the eye due to the combination of biological barriers (cornea and sclera), ocular drainage, and systemic absorption^50–53^. Such limited retinal penetration was also observed in the current study, with the difference in protection seen between the two modes of treatment indicating that, after adjusting for the dosage given per day, a 96% loss in levodopa effectiveness occurs when administered as an eye drop. This would suggest only 4% of the topical solution was available for use by the retina. However, even at these lower retinal penetration levels, topically applied levodopa was still highly effective at inhibiting the development of LIM.

Both intravitreal and topical application of levodopa were observed to inhibit the development of LIM in a similar dose-dependent manner to that observed previously for FDM^27^. This would suggest that the development of both forms of experimental myopia involve reduced dopaminergic activity. This is in accordance with a number of previous studies that have reported diminished retinal dopamine levels in both paradigms, with the majority of these analyses undertaken in chicks^11,13,16,54^. Similarly, both FDM and LIM can be inhibited by the administration of dopaminergic agonists such as apomorphine^11,21,23^ and quinpirole^19,21^, whilst the protection afforded by diffuser- or lens-removal can be blocked by the administration of dopaminergic antagonists^19,21^. The responses to levodopa treatment observed here further indicate the presence of functional similarities between FDM and LIM in response to dopaminergic manipulation. However, there are reported inconsistencies in the role of dopamine in the development of LIM, with levels reported to be unaffected by LIM in two previous studies in chicks and guinea pigs^37,38^, while the dopaminergic agonist apomorphine has been reported to affect FDM but not LIM^37^.

In accordance with the mechanism by which levodopa is hypothesised to slow ocular growth (increased dopamine release), we have previously shown that intravitreal application of levodopa increases dopamine synthesis and release within the eye during the induction of FDM^27^. To complement these findings, we show here that the protective effects of levodopa against both FDM and LIM can be abolished by co-administration with the D2-like receptor antagonist spiperone, but not the D1-like receptor antagonist SCH-23390. This confirms that the protective effects of levodopa in both models of experimental myopia are driven by dopaminergic activation of the D2-like receptor family. This aligns with work undertaken in chicks^11,19–21,34,46^ and tree shrews^22^, which has demonstrated a D2-dependent mechanism for the dopaminergic inhibition of experimental myopia. Work in tree shrews has further suggested that, of the D2-like receptor family, the D_4_ receptor subtype is critical for protection against myopia^22^. However, this D2-like receptor driven protection does not appear to be consistent across all animal models of myopia, with the Rodentia family (guinea pigs^47^ and mice^48^) demonstrating protection through a D1-like receptor mechanism. Interestingly, in mice, activation of D2-like receptors has even been postulated to be involved in myopic growth^48^, suggesting the presence of opposing actions of dopamine via the two receptor families, a phenomenon not seen in the other species studied thus far. However, despite these species’ differences in receptor mechanism, agreement remains around the critical role that retinal dopamine plays in the modulation of eye growth.

This study demonstrates, in conjunction with our previous work, the efficacy and mechanism of action by which levodopa inhibits both major forms of experimental myopia. Building on this work, future studies will look in more detail as to how increased dopamine release, through administration of levodopa, inhibits ocular growth at a biochemical level. Importantly, understanding the cellular targets of dopamine, and their location within the eye, is critical to further understanding its mechanism of action. Furthermore, it would be valuable to examine how levodopa treatment influences choroidal thickness, which is now a primary biometric measurement for human myopia, and to investigate whether differences are seen between FDM and LIM with respect to choroidal changes. Finally, critical to such animal work is to understand if and how these findings translate to human myopia. An important first step in understanding the translatability of such findings is that the two major forms of experimental myopia are similarly inhibited by levodopa administration, suggesting some level of conservation in the underlying growth mechanism.

## Conclusion

Here we show that levodopa administration, be it through intravitreal injection or topical eye drops, can retard ocular growth and significantly inhibit the development of LIM in a dose-dependent manner. Furthermore, levodopa’s protection against the development of LIM follows a similar dose-dependent pattern to that observed previously in levodopa-based protection against FDM. Finally, the protective effects of levodopa against both FDM and LIM can be abolished by co-administration with the dopamine D2-like antagonist spiperone, but not the D1-like antagonist SCH-23390, confirming that levodopa elicits its protective effects through the retinal dopaminergic system via a D2-like receptor dependent mechanism.

## Methods

### Animal housing

As previously described^27^, day-old male White-Leghorn chickens were obtained from Barter & Sons Hatchery (Horsley Park, NSW, Australia). Chicks were kept in temperature-controlled rooms and given five days to adjust to their surroundings before all experiments commenced (6 days of age). Chicks had access to unlimited amounts of food and water and were kept under normal laboratory lighting (500 lx, fluorescent lights) on a 12:12 h light:dark cycle with lights on at 9am and off at 9 pm. The experiments using animals were approved by the University of Canberra Animal Ethics Committee under the ACT Animal Welfare Act 1992 (project number CEAE 16–05) and conformed to the ARVO Statement for the Use of Animals in Ophthalmic and Vision Research.

### Myopia induction

Myopia was induced by placing either a translucent diffuser (FDM) or negative lens (− 10D, LIM) over the treated (left) eye as previously described^55^. For both paradigms, the left eye served as the experimental eye, while the right eye remained untreated and served as a contralateral control eye. Diffusers and lenses were first fitted immediately following initial drug treatments. Lenses and diffusers were briefly removed each morning before ‘lights on’ for cleaning.

### Standard experimental structure and measurement of ocular parameters

Following our previous experimental structure^27^, for all experiments chicks were given a 10 μL intravitreal injection once daily (9am, using a 30-gauge needle (Terumo) fitted to a Hamilton syringe (100 µL capacity)), or two 40 μL topical eye drops twice daily (9am and 1:30 pm), of levodopa to their diffuser- or lens-treated eye for a period of four days. For intravitreal administration, chicks were anaesthetised under light isoflurane (5% in 1 L of medical grade oxygen per minute, Veterinary Companies of Australia, Kings Park, NSW, Australia) using a vaporiser gas system (Stinger Research Anaesthetic Gas Machine (2,848), Advanced Anaesthesia Specialists, Payson, Arizona, USA).

For all drug preparations (Table 1), levodopa (Sigma Aldrich, D9628) was dissolved fresh in a solution containing 0.1% w/v ascorbic acid in 1 × phosphate-buffered saline (PBS) as outlined previously^27^. Immediately prior to administration, the pH of the levodopa solution was adjusted to 5.5. For experiments using dopaminergic antagonists, spiperone (Sigma Aldrich, S7395) or SCH-23390 (Sigma Aldrich, D054) was added to the above levodopa solution.

For all experiments, refraction, anterior chamber depth, lens thickness, vitreal chamber depth and axial length were measured on day one (prior to the commencement of experiments) and day four (2 h following morning drug administration) as previously described^27^. Refraction measurements for both treated (left) and contralateral control (right) eyes were taken using automated infrared photoretinoscopy (system provided courtesy of Professor Frank Schaeffel, University of Tuebingen, Germany) with refractive values representing the mean spherical equivalent of 10 measurements per eye. For axis alignment, the Purkinje image was centred within the pupil to obtain the correct refractive axis. Illumination levels within the room held at less than 5 lux to avoid light reflections in the pupil arising from aberrant sources. Axial length was measured, on chicks anesthetised as above, using A-scan ultrasonography (Biometer AL-100; Tomey Corporation, Nagoya, Japan) with each scan representing the mean of 10 measurements and the average of three scans taken for each eye. No differences were observed between groups or between eyes prior to the commencement of treatment.

For 15 mM topical levodopa treatment in the dose–response curve experiment, corneal curvature (measured as the radius of curvature) was also examined following the procedure outlined in Troilo & Wallman^56^ using a keratometer (Topcon OM-4) fitted with a + 8D lens to adapt the system to the highly curved chick cornea, and calibrated by measuring curvatures of chrome balls of known diameters (2–8 mm).

### LIM dose–response curves

To establish whether the dose-dependent protective effects of levodopa against the development of FDM are preserved in LIM, chicks were randomly divided into the following treatment groups (Table 1) and treated according to the standard experimental structure outlined above:

1. Fitted with a − 10D lens, daily intravitreal injection of one of the following doses of levodopa to represent levodopa being delivered directly to the retina:
  - 0.15 mM levodopa (n = 6);
  - 1.5 mM levodopa (n = 6);
  - 15 mM levodopa (n = 6);
  - 45 mM levodopa (n = 6).
2. Fitted with a − 10D lens, twice-daily topical application of one of the following doses of levodopa as eye drops to represent a potential avenue for clinical treatment:
  - 0.15 mM levodopa (n = 8);
  - 1.5 mM levodopa (n = 8);
  - 15 mM levodopa (n = 7);
  - 45 mM levodopa (n = 8).
3. Fitted with a − 10D lens (LIM Only, n = 8);
4. Fitted with a − 10D lens, daily intravitreal injection of the vehicle solution (0.1% ascorbic acid in 1xPBS, LIM Vehicle Injections, n = 8);
5. Fitted with a − 10D lens, twice-daily topical application of the vehicle solution (0.1% ascorbic acid in 1×PBS, LIM Vehicle Drops, n = 8);
6. Age-matched untreated controls (n = 12).

As stated previously^27^, due to solubility limits, a 45 mM solution, which sits at the upper solubility limit of levodopa at pH 5.5 for the duration of drug administration, was the highest dose tested.

A power calculation was undertaken to determine the group sizes required to achieve 80% power in observing a 1D change in refraction when the standard deviation is approximately 0.5D:

$${n}_{1}=\frac{{\left({\sigma }_{1}^{2}+{\sigma }_{2}^{2}/K\right)\left({z}_{1-\alpha /2} + {z}_{1-\beta }\right)}^{2}}{{\Delta }^{2}}$$

$${n}_{1}=\frac{{\left({0.5}^{2}+{0.5}^{2}/1\right)\left(1.96 + 0.84\right)}^{2}}{{1}^{2}}$$

$${n}_{1}=4$$

To account for fluctuations in standard deviation, as well as potential dropouts due to lens removal, group sizes were increased to a minimum of n = 6 for injections and n = 9 for topical drops. Numbers in the topical group were greater due to the higher potential for dropouts as the lenses were removed for treatment more often, increasing the potential for the lens mount to fail and the animal needing to be removed from the study.

The dose-dependent effects of levodopa in LIM eyes were also retrospectively compared to previous data (following the same experimental protocol) on the dose-dependent effects of levodopa in form-deprived eyes^27^.

### Determination of dopamine receptor subtype

To establish the receptor subtype by which levodopa induced dopamine release inhibits experimental myopia, levodopa was tested in combination with an antagonist of the D1-like dopamine receptor family (SCH-23390) and an antagonist of the D2-like dopamine receptor family (spiperone) at concentrations used previously^19^. These antagonists were co-administered intravitreally with 15 mM levodopa, a dose at which experimental myopia is abolished (Table 2). Chicks were randomly divided into the following groups (Table 1) and treated according to the standard experimental structure outlined above:

1. Fitted with a − 10D lens to induce LIM, intravitreal injection of:
  - levodopa and SCH-23390 (15 mM:0.5 mM, n = 6);
  - levodopa and spiperone (15 mM:0.5 mM, n = 6);
  - levodopa alone (15 mM, n = 6).
2. Fitted with a translucent diffuser to induce FDM, intravitreal injection of:
  - Levodopa and SCH-23390 (15 mM:0.5 mM, n = 6);
  - Levodopa and spiperone (15 mM:0.5 mM, n = 6);
  - Levodopa alone (15 mM, n = 6).
3. Fitted with a -10D lens (LIM Only, n = 8);
4. Fitted with a translucent diffuser (FDM Only, n = 12);
5. Age-matched untreated controls (n = 12).

After no differences were observed between LIM only and LIM vehicle treated groups in the dose–response curve experiment, or between FDM only and FDM vehicle treated groups in our previous study^27^, vehicle treated groups were not included in this experiment.

### Statistical analysis

All values reported represent the mean ± the standard error of the mean (including outliers). Any chicks which removed their lenses or diffusers were removed from the experiments and are therefore not reported. Before analysing the effect of treatment, all data, which represented measurements from individual chickens not technical replicates, were first tested for normality and homogeneity of variance (Shapiro–Wilk test). When there was no significant variance in normality or homogeneity, the effect of treatment was analysed via a one-way univariate analysis of variance (ANOVA). When significant, ANOVAs were followed by a student’s unpaired *t*-test with Bonferroni correction for multiple testing for analysis of specific between group effects. For the retrospective analysis of levodopa’s effects against LIM compared to its dose-dependent effects against FDM seen in our previous study^27^, a multivariate analysis of variance (MANOVA) was undertaken. All analyses were undertaken in IBM SPSS Statistics package 23 with a statistical significance cut-off of 0.05.

## Data Availability

All data generated or analysed during this study are included in this published article.

## Abbreviations

DOPAC: 3,4-Dihydroxyphenylacetic acid
FDM: Form-deprivation myopia
LC–MS-MS: High performance liquid chromatography-tandem mass spectrometry
LIM: Lens induced myopia
PBS: Phosphate buffered saline
