# Author Correction: Levodopa inhibits the development of lens-induced myopia in chicks

**DOI:** 10.1038/s41598-021-94667-7

**Published:** 2021-07-27

**Authors:** Kate Thomson, Ian Morgan, Cindy Karouta, Regan Ashby

**Affiliations:** 1 Centre for Research into Therapeutic Solutions, Faculty of Science and Technology, University of Canberra, Canberra, Australia; 2 Research School of Biology, Australian National University, Canberra, Australia

Correction to: *Scientific Reports* 10.1038/s41598-020-70271-z, published online 06 August 2020

The original version of this Article contained errors. In Table 1, some of the values for “Concentration (% w/v)” and “Amount Given (mg/day)” were incorrectly reported.

In Table 1, in the ‘Concentration’ column, for Levodopa/Spiperone/Lens (line 13) and Levodopa/Spiperone/Diffuser (line 16),

“0.596/0.002”

now reads

“0.296/0.002”.

Additionally, in Table 1, in the ‘Amount Given (mg/day)’ column, for 45 mM levodopa (line 9)

“0.900”

now reads

“0.090”.

The original version of the Article has been corrected.

